# Biosilicification in monocots: Comparative analysis highlights contrasting patterns of deposition

**DOI:** 10.1002/ajb2.70074

**Published:** 2025-07-17

**Authors:** Paula J. Rudall, Jehova Lourenco, Manoj Kumar Mahto

**Affiliations:** ^1^ Jodrell Laboratory Royal Botanic Gardens, Kew Richmond TW9 3AB Surrey UK

**Keywords:** anatomy, monocots, phylogeny, phytoliths, SEM‐EDX‐EDS, silica deposition

## Abstract

**Premise:**

New insights into biomineral uptake and sequestration are important for understanding how plants grow. Some plants accumulate silica accretions in precise locations in particular cells. Among monocots, controlled biosilicification occurs in several different forms and is restricted to commelinids and orchids.

**Methods:**

We utilized energy‐dispersive x‐ray spectroscopy (EDX/EDS) mapping technology on leaf transverse sections to explore the diverse silica deposition patterns in a range of monocots. The results were evaluated using character optimizations on existing phylogenies.

**Results:**

Our optimization indicates at least two independent evolutionary origins of phytoliths among monocots, with secondary losses in some lineages. Silica that accumulates in the cell lumen occurs mostly in bundle sheath cells or epidermal cells, often associated with sclerenchyma. In Bromeliaceae and Rapateaceae, small phytoliths occur in the walls of occluded epidermal cells overlying sclerenchyma. In Dasypogonaceae, phytoliths accumulate in the lumen of epidermal cells. Cell‐wall bound silica occurs in the epidermal cells of some commelinids (Commelinaceae, Cyperaceae and Poaceae). There is a close association between silica deposition and the presence of ferulic acid, except possibly in orchids. Records of high silica concentration in leaves are not always correlated with deposition. We found no silica deposition in leaves of some aquatic commelinids, despite evidence for silica uptake and presence of ferulic acid.

**Conclusions:**

Our ongoing comparative investigations using EDX data not only extend our knowledge about biomineral inclusions in plants, but also highlight their structural and biochemical complexity. This study suggests that the diversity and relatively restricted phylogenetic distribution of monocot phytoliths is at least partly attributable to cell chemistry.

Silica that is present in soils is frequently assimilated by living plants. Soluble silicic acid enters plants via their roots and is transported through the xylem to the aerial organs, driven either by passive transpiration and dehydration or by more active modes of transport across channels (Mitani and Ma, 2005; Bauer et al., 2011; Exley, 2015). High silica accumulation characterizes some lycophytes, horsetails, eusporangiate ferns and some spermatophytes (Hodson et al., 2005; Katz, 2015; Trembath‐Reichert et al., 2015; Song et al., 2020; Tombeur et al., 2023; Pang et al., 2025). Accumulation differs considerably among taxa, indicating that it is under strong genetic regulation. Some accumulators simply maintain silicic acid in supersaturated colloidal solution, probably mostly in the xylem sap, whereas others build up localized accretions of silica, termed phytoliths (Exley, 2015). Thus, there is a clear distinction between silica accumulation and silica deposition in plant tissues. Silica accretions can persist for long periods in the substrate following the death of the plant that produced them, thereby providing characteristic markers in archaeological material and some fossil deposits, mostly from the Tertiary and especially the Quaternary periods (Sakai and Thom, 1979; Bennett, 1982; Piperno, 1988, 2006; Pearsall, 2015; Siver et al., 2025). Among land plants, silicified tissues can occur in a wide range of taxa, but phytolith accretion is perhaps most typical of ferns (including *Equisetum*) and monocots (including grasses).

Controlled silicification leading to phytolith assembly occurs in specific tissues at particular target sites, focused initially on the primary cell walls (Kumar et al., 2017; Wani et al., 2023). Silica can be deposited either in the secondary cell wall or within the membrane‐bound lumens of localized idioblasts, forming granules or discrete bodies of solid hydrated opaline silica (silicon dioxide, SiO_2_·*n*H_2_O). Most deposition sites occur either in the bundle sheath or the epidermis, often associated with sclerenchyma (Lanning and Eleuterius, 1987). Our investigation builds upon considerable recent progress in understanding the biology of silica and complex associated processes (Tripathi et al., 2020). Contrasting deposition pathways are either scaffolded by organic materials associated with cell walls or grow by hyperaccumulation in a progressively supersaturated vacuole in the cell lumen (Guerriero et al., 2016; Soukup et al., 2017; Bokor et al., 2019).

To examine the different types of silica deposition that occur in the tissues of monocot photosynthetic organs, we used energy‐dispersive x‐ray spectroscopy (EDX or EDS) on transverse sections. We focused on monocots because some taxa are already known to be characterized by distinctive phytoliths (Figure 1). The consistent presence of silica bodies in some monocot families—and their reliable absence from others—have been documented since the late 19th century (early studies were summarized by Kohl, 1889). However, the phylogenetic significance of silica bodies was not explicitly recognized until the late 20th century, when monocot classification was transformed, initially by character‐based studies (Dahlgren and Rasmussen, 1983; Dahlgren et al., 1985) and subsequently by phylogenetic and phylogenomic analyses (e.g., Soltis et al., 2000; Givnish et al., 2018; Timilsena et al., 2022; Zuntini et al., 2024). The resulting consensus tree identified a series of lineages of early‐divergent and lilioid monocots, and a robust and well‐supported commelinid clade (e.g., Givnish et al., 2018). Silica has been reported in all the commelinid orders, which include many tropical and temperate taxa (e.g., bromeliads, commelinas, gingers, grasses, palms, and sedges), but is absent from all other monocots except orchids (Prychid et al., 2004). Thus, the occurrence of silica bodies in orchids appears anomalous in this context because orchids have been consistently placed within the non‐commelinid order Asparagales in all recent phylogenetic and phylogenomic analyses, most commonly as sister to all other Asparagales (e.g., Givnish et al., 2018; Timilsena et al., 2022; Wang et al., 2024).

**Figure 1:**
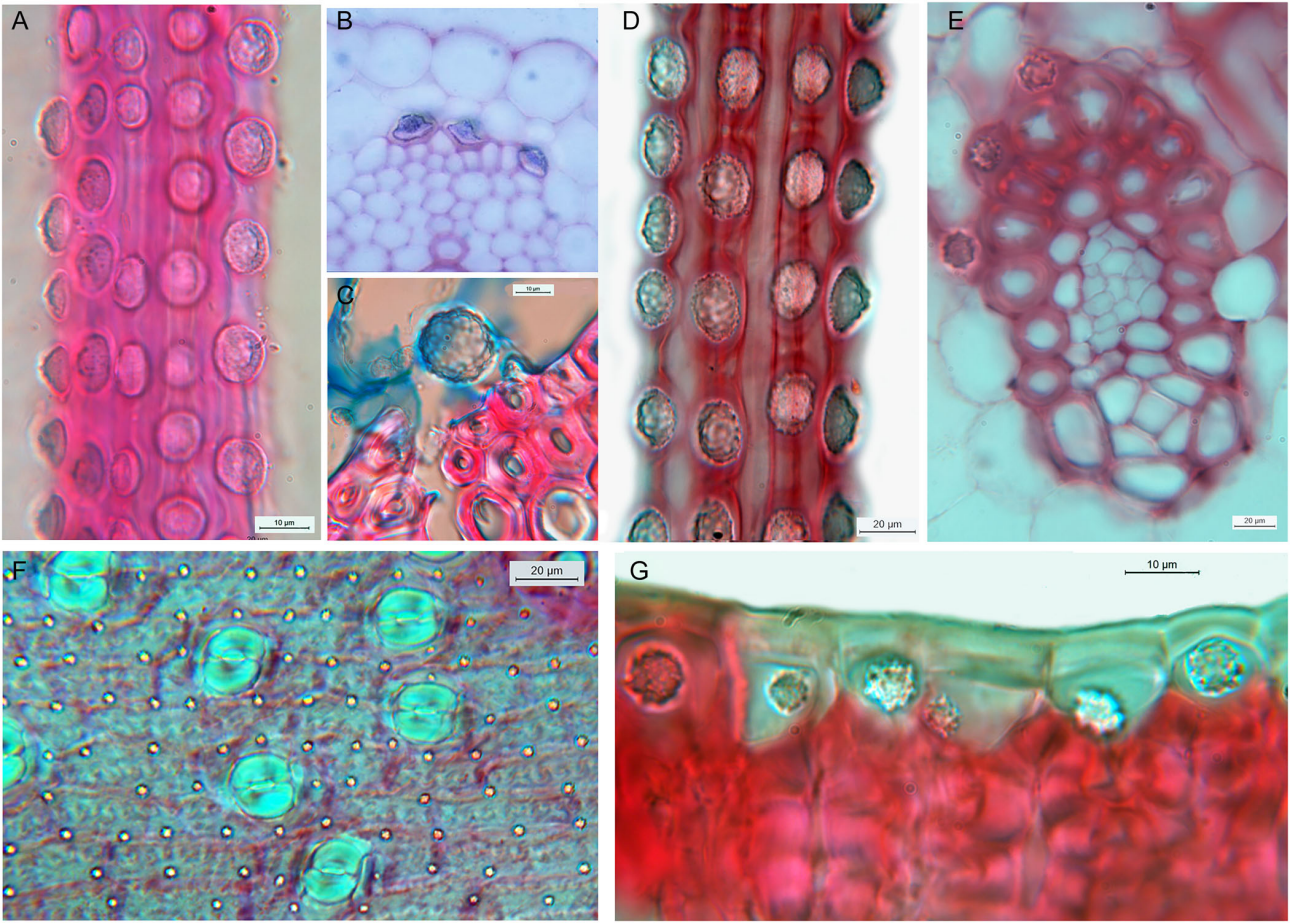
Light micrographs of silica bodies in idioblasts in either the vascular bundle sheath (A–E) or epidermis (F, G) in permanent microscope slides held at the Royal Botanic Gardens, Kew. (A) *Apostasia nuda* R. Br. (Orchidaceae–Apostasioideae, Asparagales), longitudinal section (LS) of vascular bundle. (B) *Cephalanthera pallens* Hohen. (Orchidaceae– Epidendroideae, Asparagales), transverse section (TS). (C) *Eria javanica* (Sw.) Blume (Orchidaceae– Epidendroideae, Asparagales), TS of leaf. (D) *Chamaedorea cataractarum* Mart. (Arecaceae–Arecales), LS of vascular bundle. (E) *Cocos nucifera* L. (Arecaceae–Arecales), TS of vascular bundle. (F) *Navia nubicola* L.B. Sm. (Bromeliaceae–Poales), surface view. (G) *Kingia australis* R. Br. (Dasypogonaceae–Dasypogonales), TS. Scale bars = 10 µm.

Most previous EDX‐based analyses of silica in monocots have centered on the grass family Poaceae (e.g., Soni et al., 1972; Terrell and Wergin, 1981; Kaufman et al., 1970, 1985; Zexer and Elbaum, 2020; Zexer et al., 2023). Our investigation differs from previous studies using EDX, not only in sampling a phylogenetically broad range of taxa, but also in examining silica distribution among tissues within the leaf or stem, rather than merely on the surface. Consistent differences in silica localization indicate diverse mechanisms for controlled deposition. Possible factors in controlled cell‐wall silicification include cell‐wall polymers such as lignin, callose, and ferulic acid (Zexer and Elbaum, 2020, Zexer et al., 2023). Thus, examining co‐localization of silica with other compounds in plant tissues could help to elucidate secondary functions. Evaluating our findings in a perspective that is both phylogenetically broad and essentially comparative can enhance our interpretation of the evolution of silica deposition in land plants.

## MATERIALS AND METHODS

### Sample collection

We focused primarily on the well‐documented silica‐bearing monocot clades, the commelinids and orchids. For comparison, we also tested two other monocot groups: (1) some putative orchid relatives among early‐divergent Asparagales (Asteliaceae and Boryaceae) and (2) *Acorus*, which represents the sister taxon to all other monocots in recent molecular phylogenetic analyses. Most analyzed species were sampled from among the extensive living collections of the Royal Botanic Gardens, Kew (United Kingdom), both from plants growing in planted areas of large greenhouses and in the open air, in a wide range of soil conditions. The remainder were sampled from Kew's spirit collections. All samples were taken from mature specimens, and they were sampled as far as possible from central regions of the leaf blade rather than the leaf sheath. Fresh material was fixed in 70% v/v ethanol before sectioning. Materials examined are listed in Table 1.

**Table 1:** List of species and material examined for EDX/EDS analysis. Most plant material was collected either from the living collections at the Royal Botanic Gardens, Kew (HK), with their accession numbers if available (s.n. = number absent), or from fixed material held in Kew's spirit collections (SC).

Taxonomic grouping	Species	Source
**Commelinid monocots**		
Arecaceae	*Trachycarpus fortunei* (Hook.) H.Wendl.	HK s.n.
	*Bismarckia nobilis* Hildebrandt & H.Wendl.	HK 2006‐1657
Dasypogonaceae	*Calectasia cyanea* R.Br.	PJR 37
Poales, Bromeliaceae	*Aechmea recurvata* (Klotzsch) L.B.Sm.	HK 1972‐321
	*Aechmea victoriana* L.B.Sm.	HK 1972‐3422
	*Fascicularia bicolor* (Ruiz & Pav.) Mez	HK 2000‐2596
Poales, Cyperaceae	*Carex depauperata* Curtis ex Woodw.	HK 1954‐19002
	*Carex testacea* Sol. ex Boott	HK 2009‐2048
	*Carex secta* Boott	HK 2006‐1728
	*Mapania palustris* (Hassk. ex Steud.) Fern.	HK 2013‐1499
	*Schoenoplectus lacustris* (L.) Palla	HK 2002‐69
Poales, Flagellariaceae	*Flagellaria indica* L.	PJR 112
Poales, Joinvilleaceae	*Joinvillea plicata* (Hook.f.) Newell & B.C.Stone (stem)	NSW 612730
Poales, Juncaceae	*Juncus xiphioides* [stem]	HK 1997‐5610
Poales, Poaceae	*Stipa gigantea* Lag.	HK 2009‐795
	*Glyceria maxima* (Hartm.) Holmb.	HK 1969‐19071*3
Poales, Rapateaceae	*Rapatea xiphoides* Sandwith	SC 16627
Poales, Restionaceae	*Restio* sp. [stem]	Linder 17.8.87
Poales, Typhaceae	*Typha minima* Funck	HK 2012‐519*2
Poales, Xyridaceae	*Xyris longiscapa* L.A.Nilsson [stem]	HK
Zingiberales, Hanguanaceae	*Hanguana* sp.	PJR 111
Zingiberales, Strelitziaceae	*Strelitzia* sp.	HK s.n.
Zingiberales, Zingiberaceae	*Etlingera hemisphaerica* (Blume) R.M.Sm.	HK
Commelinales, Commelinaceae	*Pollia macrophylla* (R.Br.) Benth.	HK 1988‐224
	*Pollia condensata* C.B.Clarke	HK 1993‐3114
	*Tradescantia spathacea* Sw.	HK 1979‐2389
Commelinales, Pontederiaceae	*Pontederia caudata*	HK 1969‐50051
**Non‐commelinid monocots**		
Acorales, Acoraceae	*Acorus gramineus* Aiton	HK 1969‐17999
Asparagales, Asteliaceae	*Astelia fragrans* Colenso	HK 2006‐1890
Asparagales, Boryaceae	*Borya sphaerocephala* R.Br. [bract]	SC 909
Asparagales, Orchidaceae	*Dendrobium* × *delicatum* (*D. kingeanum* × *D. speciosum*)	HK 1980‐4430
	*Bletilla striata* (Thunb.) Rchb.f.	HK 2006‐561
	*Vanilla polylepis* Summerh.	HK 1973‐13709

### Energy‐dispersive x‐ray spectroscopy (EDX or EDS) analysis

To effectively visualize silica deposits, we used backscattered electron imaging, which provides surface topography, and complementary EDX analysis for elemental composition. Thick transverse sections of photosynthetic organs—commonly leaves but occasionally stems—were hand‐cut using a single‐edged razor blade. The sections were then air‐dried, mounted onto stubs using carbon tabs, with the cut surface uppermost, and carbon‐coated with a Quorum Technologies (Lewes, UK) Q150T sputter coater using pulse rod evaporation. They were examined using a Hitachi High‐Tec (Krefeld, Germany) Regulus 8230 Scanning Electron Microscope (FEG‐SEM) fitted with a cold‐field emission gun optimized for high‐resolution imaging (in this case at 10 kV). We used an Oxford Instruments (Abingdon, UK) Ultim Extrem detector combined with the AZtecLive (Sutton, UK) software platform version 6.2. We employed the mapping technique, which allows simultaneous examination of sample morphology and elemental distribution. This technology enabled us to present relatively high‐definition tissue regions with different elements in different colors. To provide optimal contrast, we selected red for silica, white for oxygen and blue for calcium (and green for potassium). The percentage silica results represent the relative abundances of each analyzed element in the current field of view of the sample. Each element has a unique set of energy peaks corresponding to the x‐ray emissions it produces. The positions of these peaks on the spectrum identify which elements are present, and the intensity of each peak is proportional to the concentration of the corresponding element. Positive records for silica deposition are those with clearly delimited silica deposits and/or a distinct pattern of deposition (e.g., in the bundle sheath cells or epidermal walls). We treat as negative cases where no obvious deposition pattern is observable and silica is present at relatively low levels (e.g., below ~1% in an entire transverse leaf section), which can represent a background reading (but exceptions are outlined in the results).

### Light microscopy

For light microscopy (LM), we used the extensive collection of permanent microscope slides located at the Royal Botanic Gardens, Kew (Figure 1). Many of these slides were prepared for the volume series *Anatomy of the Monocotyledons*, used as a source of published comparative data (e.g., Cutler, 1969; Tomlinson, 1961, 1969; Metcalfe, 1971; Stern, 2014). Photomicrographs were captured using a Leica DM LB100T light microscope fitted with a Zeiss AxioCam HRc digital camera.

### Character optimizations

Character optimizations of silica deposition and cell‐wall‐bound ferulic acid in monocots were performed using Mesquite version 3.81 (Maddison and Maddison, 2019–2023) and the data in Table 2. We used a composite tree topology to summarize the relevant relationships among monocots, based on the following papers: Givnish et al. (2018) for higher‐level relationships in monocots (but note that other analyses place Asparagales and Liliales as a sister pair, and relationships among the commelinid orders can differ), Wang et al. (2024) for Asparagales (but note that others place orchids among the astelioids), and Perez‐Escobar et al. (2021) for orchid subfamilies.

**Table 2:** Summary of comparative data for silica and ferulic acid in monocots, used to construct character optimizations in Figure 2. References: 1–11 silica data, 12–13 cell‐wall data (other references cited in text). (1) This paper. (2) Prychid et al. (2004) and literature cited therein. (3) Sandoval‐Zapotitla et al. (2010). (4) Brightly et al. (2024). (5) Tomlinson et al. (2011). (6) Aerne‐Hains and Simpson (2017). (7) Pereira et al. (2011). (8) Versieux et al. (2010). (9) Estelita and Rodrigues (2012). (10) Rudall et al. (2014). (11) Kumar et al. (2017). (12). Chen and Smith (2013). (13) Harris and Hartley (1980). (14) Harris et al. (1997). (15) Rudall and Caddick (1994). (16) Huisman et al. (2018). Characters: (A) Silica presence/absence (silica absent or present only as unlocalized background trace = 0; silica present = 1). (B) Silica location (absent = 0; present mostly or exclusively in bundle sheath = 1; mostly or exclusively epidermal = 2). (C) Location in cells (absent = 0; present in cell lumens = 1; present in epidermal cell walls = 2). (D) Ferulic acid (absent = 0; present = 1).

			Characters			
Order	Family	References	A	B	C	D
**Commelinid monocots**						
Arecales	Arecaceae	1,2,4,5,13,16	1	1	1	1
Dasypogonales	Dasypogonaceae (incl. *Calectasia*)	1,2,15	1	(1)2	1	1
Commelinales	Commelinaceae	1,2,13,14	0,1	0,2	0,1,2	1
	Haemodoraceae	2,5,14	0,1	0,1,2	0,1	1
	Philydraceae	2,13	0	0	0	1
	Pontederiaceae	2,13	0	0	0	1
Poales	Anarthriaceae	2	0	0	0	?
	Bromeliaceae	1,2,7,8,13	1	(1)2	1	1
	Cyperaceae	1,2,7,13	1	(1)2	1,2	1
	Ecdeiocoleaceae	2	1	?	?	?
	Eriocaulaceae	13	0	0	0	1
	Flagellariaceae	1,2,13	1	1	1	1
	Joinvilleaceae	2,13	(1)	?	?	1
	Juncaceae	2,13	0	0	0	1
	Mayacaceae	12,14	0	0	0	1
	Poaceae	1,2,10,11,13	1	2	1,2	1
	Rapateaceae	1,2,13	1	(1),2	1	1
	Restionaceae s.l.	1,2,13	1	1	1	1
	Thurniaceae	2,13	1	2	1	1
	Typhaceae s.l.	1,2,13	0	0	0	1
	Xyridaceae	1,2,13	0	0	0	1
Zingiberales	Cannaceae	2,12,13	1	1	1	1
	Costaceae	2,12	1	1	1	?
	Hanguanaceae	1,2,14	1	1 +	1	1
	Heliconiaceae	2,12	1	1	1	?
	Lowiaceae	2,12,13	1	1	1	1
	Marantaceae	2,12,13	1	1 +	1	1
	Musaceae	2,12,13	1	1	1	1
	Strelitziaceae	2,12,13	1	1	1	1
	Zingiberaceae	2,12,13,14	1	1,2	1	1
**Non‐commelinid monocots**						
Asparagales	Asteliaceae	1,2,14	(0)	0	0	01
	Boryaceae	1,2	(0)	0	0	?
	Orchidaceae	1,2,3,13	0,1	0,1	1	0
	Other families	2,13,14	0	0	0	0
Liliales	8 families	2,13	0	0	0	0
Dioscoreales	3+ families	2,13	0	0	0	0
Pandanales	5 families	2,13	0	0	0	0
Petrosaviales	Petrosaviaceae	2	0	0	0	?
Alismatales	12 families	2,13	0	0	0	0
Acorales	Acoraceae	1,2,13	(0)	0	0	0

## RESULTS

**Figure 2:**
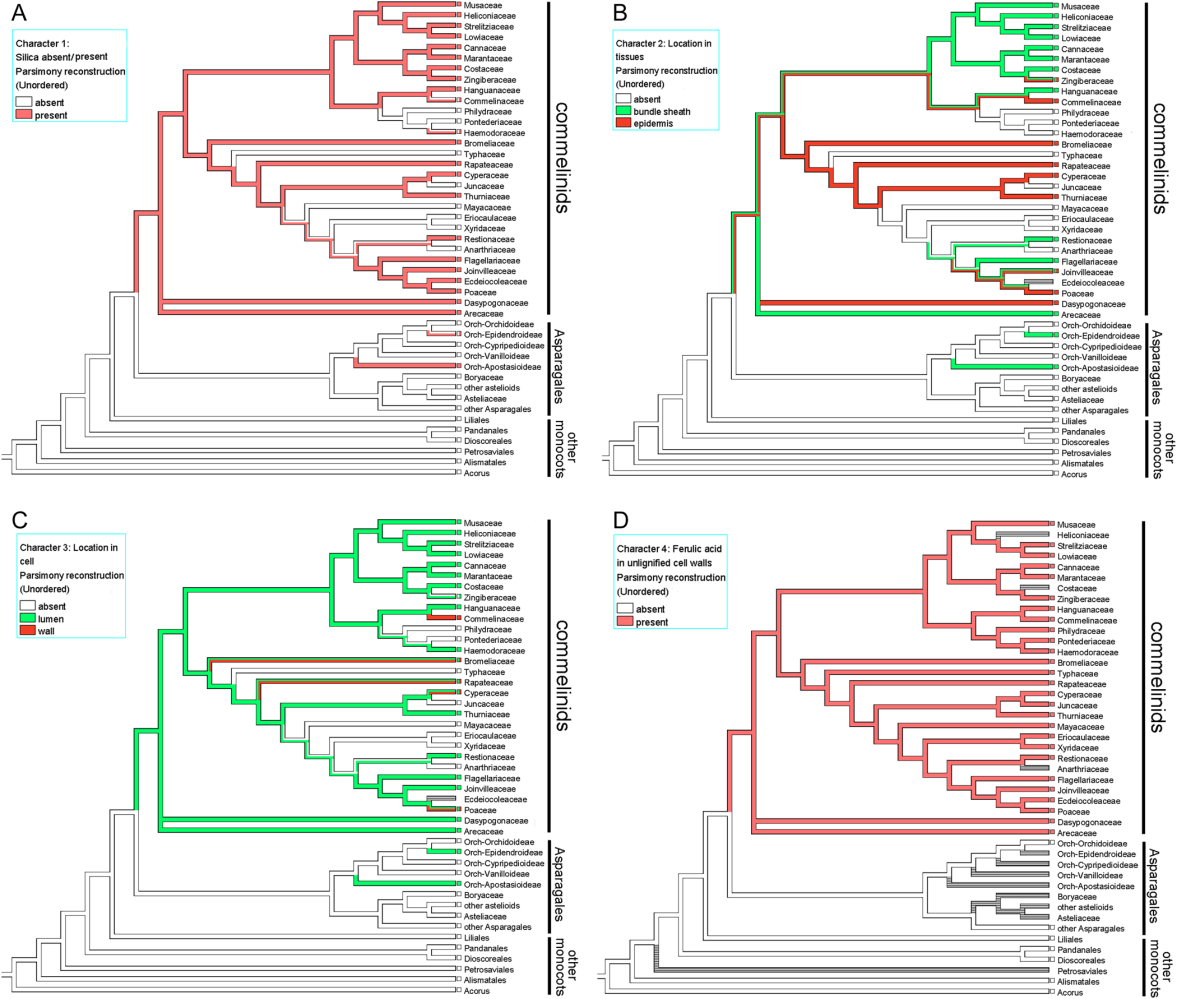
Character optimizations in monocots. A–D, see text for further details.

**Figure 3:**
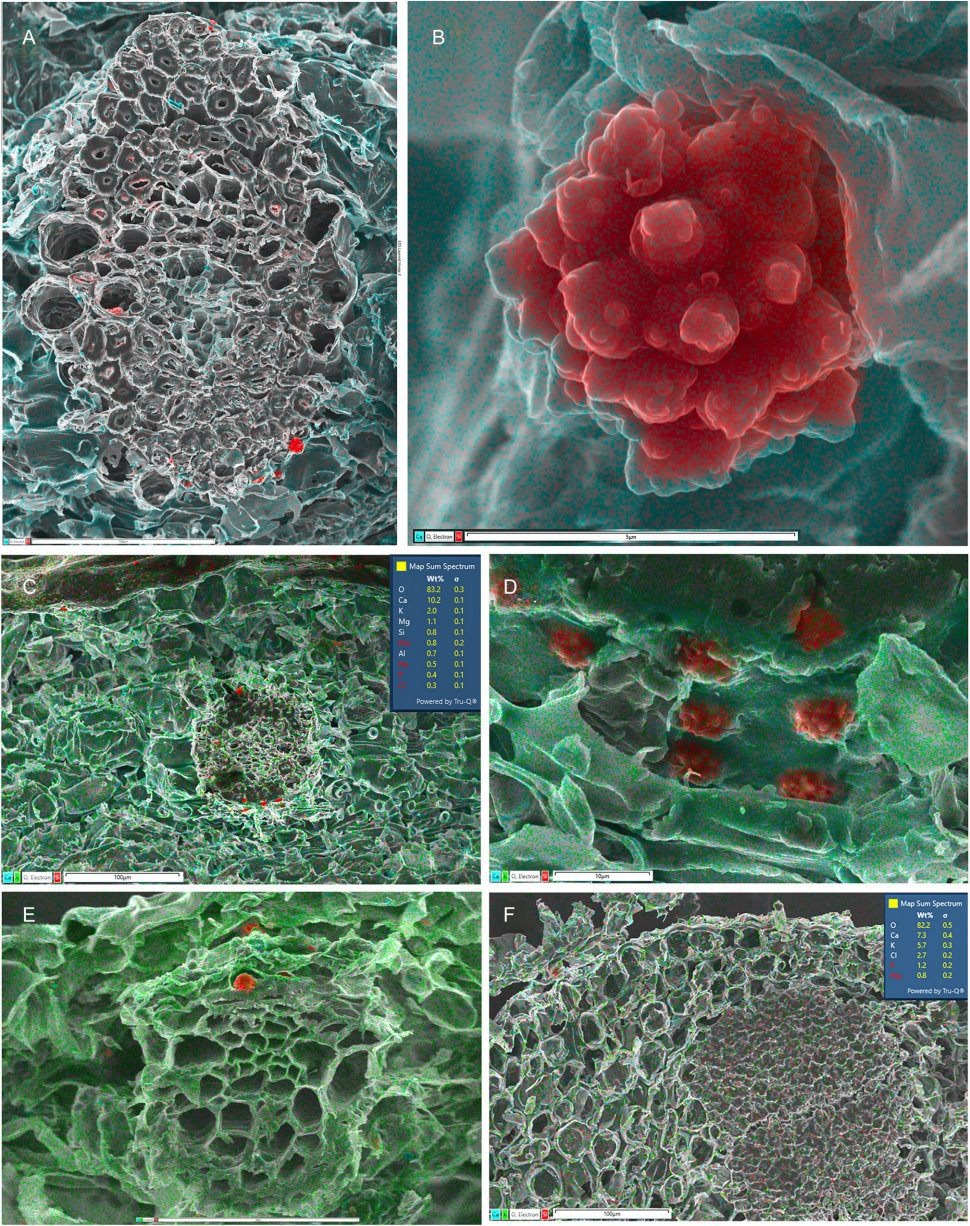
Silica mapping (in red) in leaf transverse sections of Asparagales. (A–E) Orchidaceae–Epidendroideae, silica deposits mostly restricted to bundle sheath cells (with fragments elsewhere), absent from epidermis. (A–D) *Dendrobium* ×*delicatum* (Epidendroideae–Orchidaceae), (A) entire vascular bundle with phytoliths in bundle sheath cells, (B) single phytolith inside bundle sheath cell, (C: silica 0.8%) leaf section, and (D) group of phytoliths on bundle sheath surface. (E) *Bletilla striata* (Epidendroideae–Orchidaceae), phytoliths mostly restricted to bundle sheath cells. (F) *Astelia fragrans* (Asteliaceae–Asparagales), showing part of epidermis and vascular bundle; no silica detected throughout leaf. Map sum spectra of elements shown in C and F (inset). Scale bars: A, C = 100 µm; B = 5 µm; D = 10 µm, E = 50 µm; F = 100 µm.

**Figure 4:**
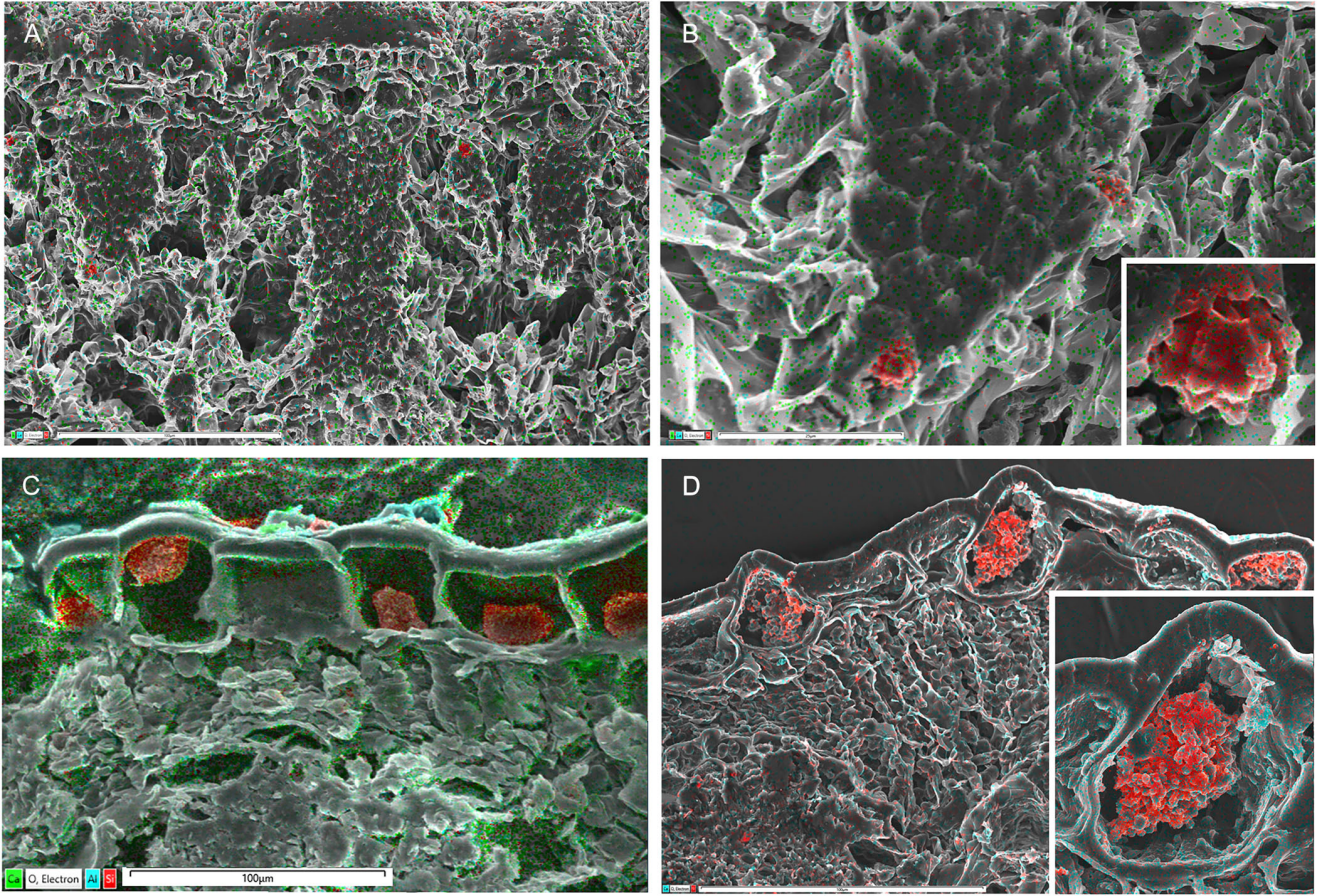
Silica mapping (in red) in leaf transverse sections of Arecaceae (palms) and Dasypogonaeae. (A, B) *Bismarckia nobilis* (Arecaceae, Arecales), silica present in bundle sheath cells, absent from epidermis, mainly associated with sclerenchyma (3.2% in A; detail of phytolith inset in B). (C, D) *Calectasia cyanea* (Dasypogonaceae, Dasypogonales), silica present in epidermal cells (5.6% in C; detail of phytolith inset in D). Scale bars: A, C, D = 100 µm; B = 25 µm.

**Figure 5:**
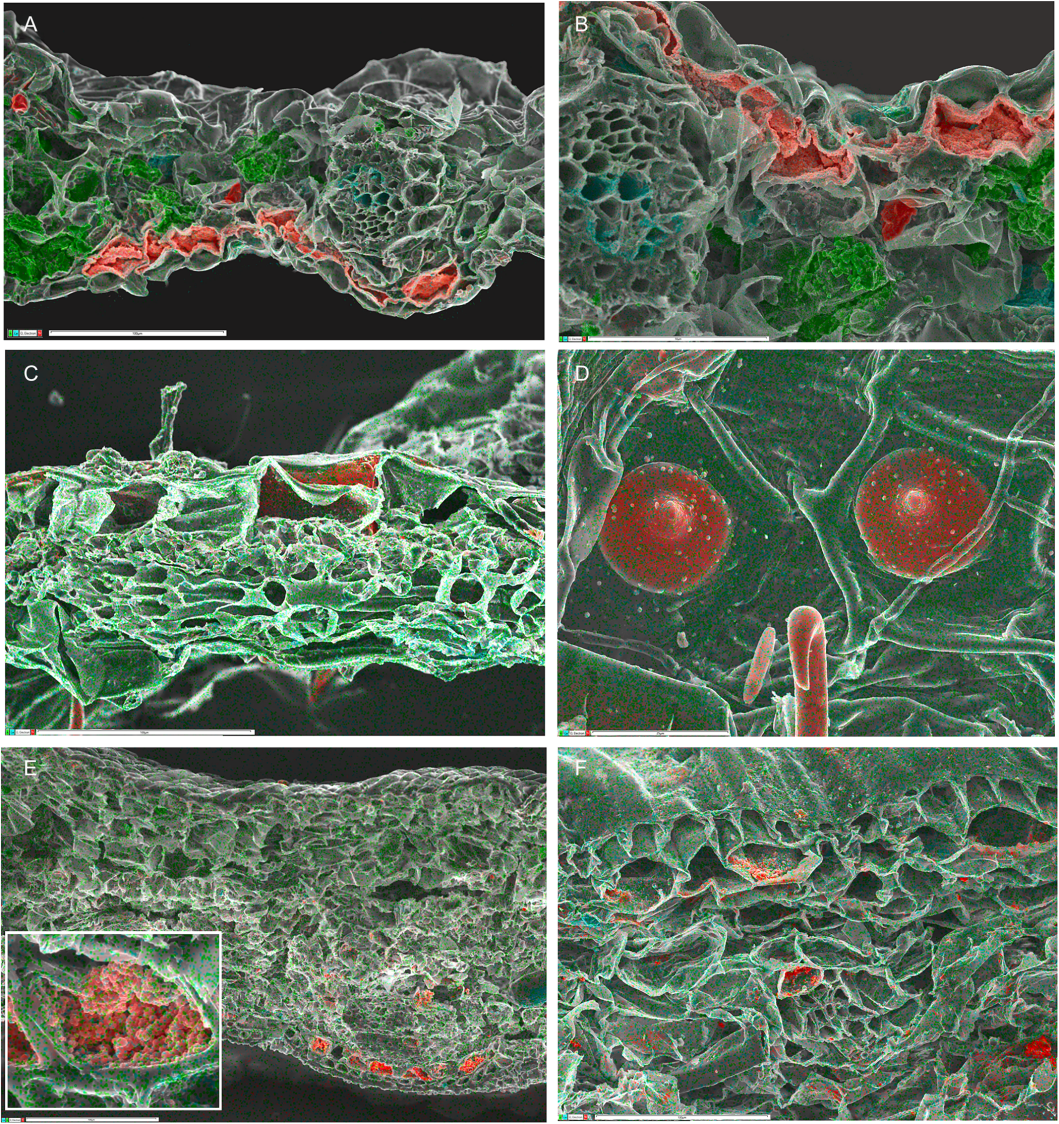
Silica mapping (in red) in leaf transverse sections of Commelinales and Zingiberales. (A, B) *Pollia macrophylla* (Commelinaceae, Commelinales) (silica 8.7% in A). (C, D) *Pollia condensata* (Commelinaceae, Commelinales) (silica 9.9% in C, surface view in D). (E) *Strelitzia* sp. (Strelitziaceae, Zingiberales) (silica 1.5% in this field of view; detail of phytolith inset). (F) *Hanguana* sp. (Hanguanaceae, Zingiberales) (silica 3.2%).

**Figure 6:**
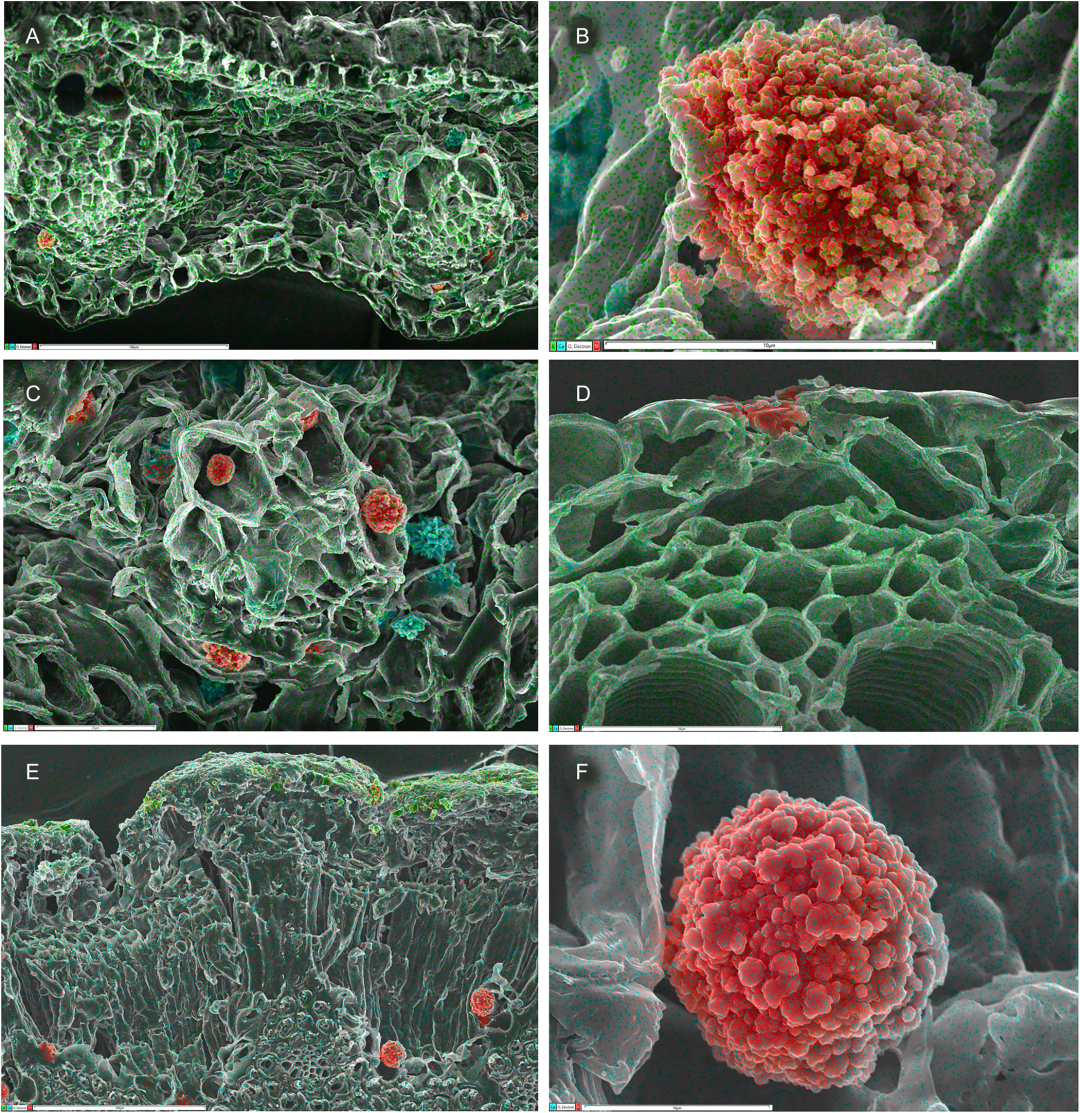
Silica mapping (in red) in leaf and stem transverse sections of three families of Poales. (A–C) *Flagellaria indica* (Flagellariaceae), phytoliths present in bundle sheath cells, absent from epidermis. (A) Leaf transverse section, with silica 2.4%. (B) Single phytolith. (C) Vascular bundle, with phytoliths restricted to bundle sheath cells and calcium oxalate crystals present in surrounding mesophyll cells. (D) *Glyceria maxima* (Poaceae), phytoliths restricted to epidermal cells overlying vascular bundles (silica 3.1% in this view). (E, F) *Restio* sp. (Restionaceae), phytoliths present in bundle sheath cells (silica 6.7% in F), and single phytolith. Scale bars: A, E = 100 µm; B, F = 10 µm; C, D = 25 µm.

**Figure 7:**
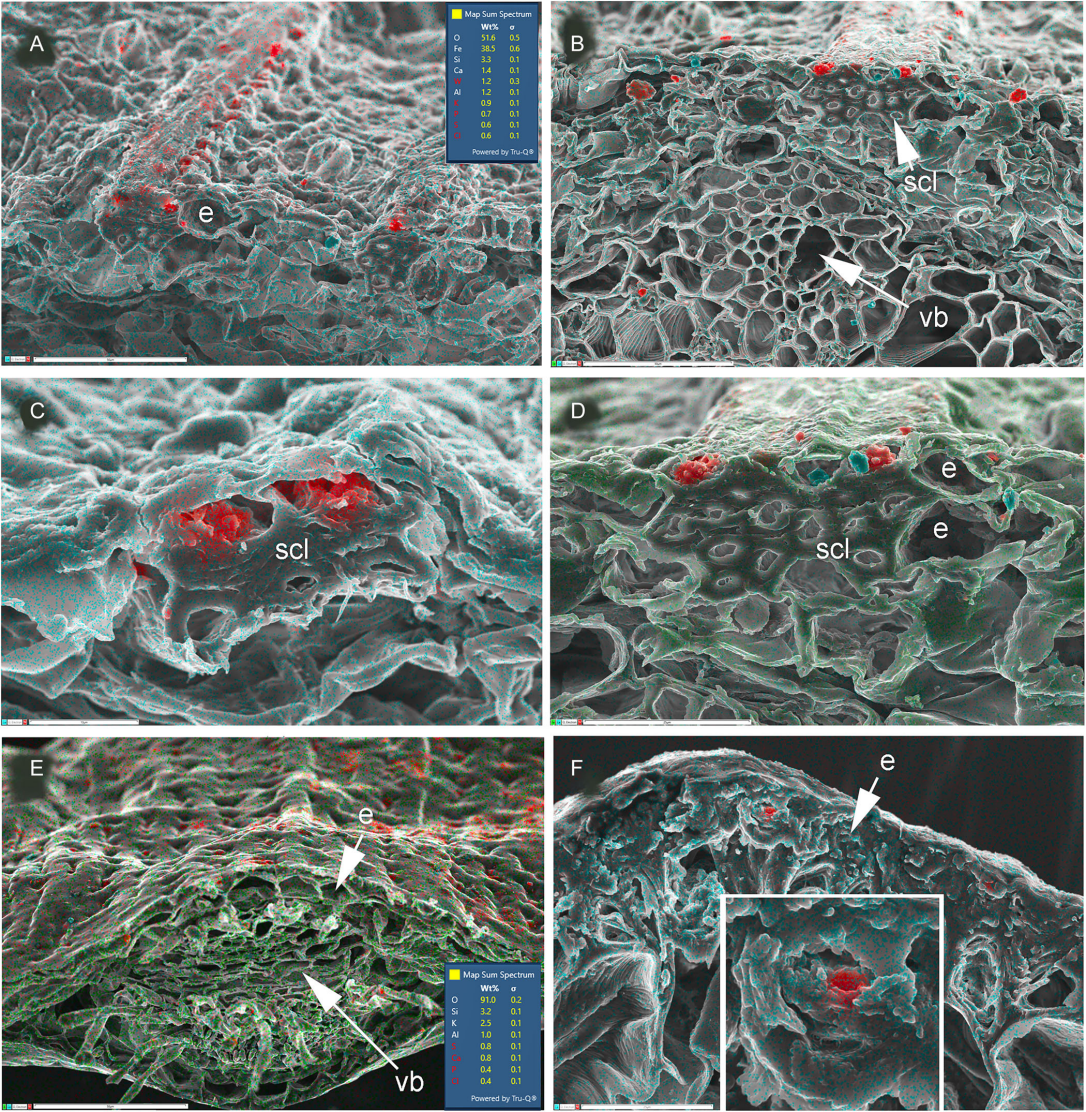
Silica mapping (in red) in leaf transverse sections of two early‐divergent families of Poales (Bromeliaceae and Rapateaceae) showing silica mostly restricted to epidermal cells. (A–D) *Rapatea xiphoides* (Rapateaceae); small epidermal silica bodies associated with hypodermal sclerenchyma bundles. (E) *Aechmea victoriana* (Bromeliaceae), small epidermal silica bodies (silica 3.2%). (F) *Fascicularia bicolor* (Bromeliaceae), small silica bodies in occluded epidermal cells, with detail inset. e = epidermis, scl = sclerenchyma, vb = vascular bundle. Scale bars: A, B = 50 µm; C = 10 µm; D, F = 25 µm; E = 100 µm.

**Figure 8:**
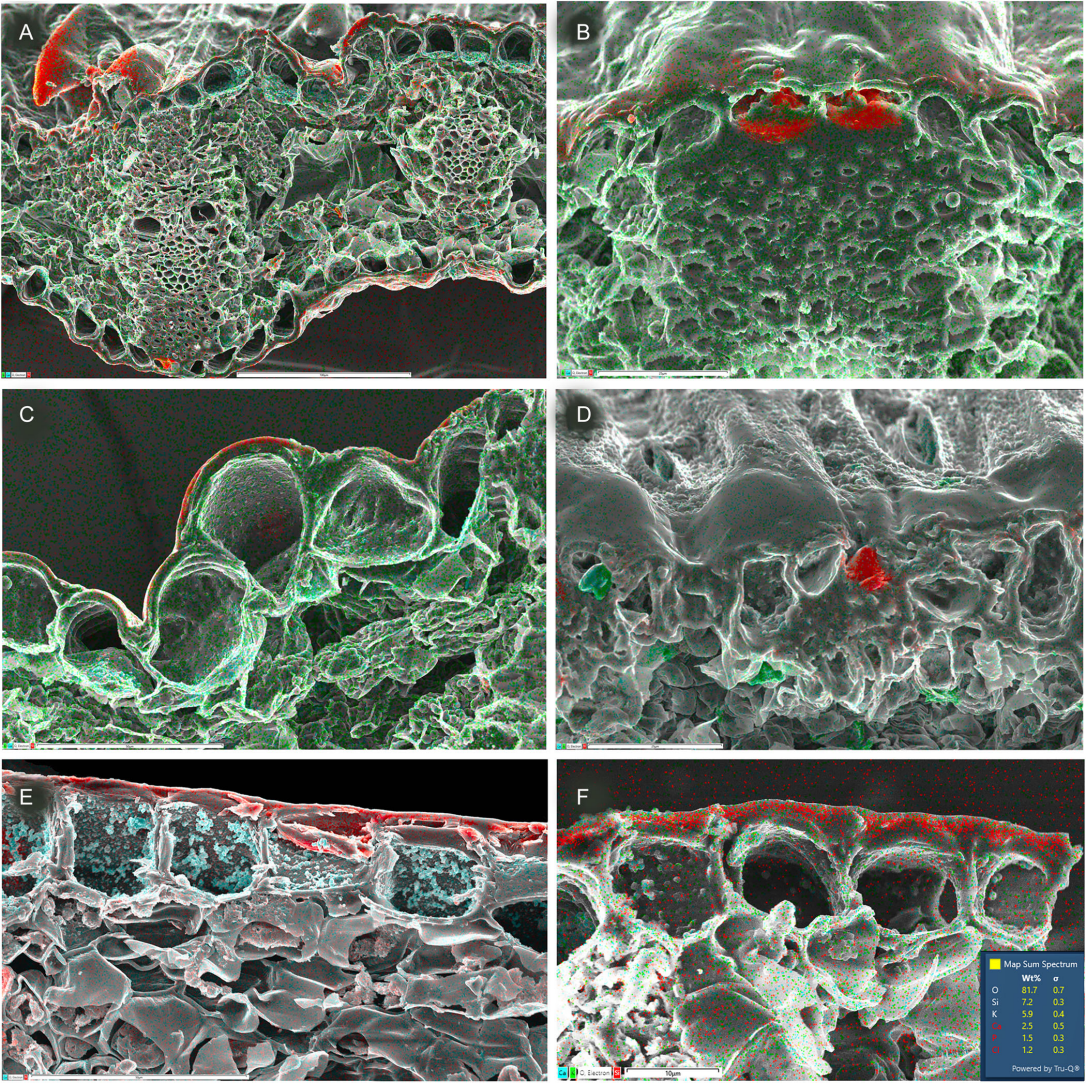
Silica mapping (in red) in leaf transverse sections of sedges (Cyperaceae–Poales). (A–C) *Carex depauperata*, showing leaf transverse section with silica mainly in epidermis, including silicified trichomes (A), conical phytoliths over hypodermal sclerenchyma (A, B), and silica in outer epidermal wall (A, C). (D) *Schoenoplexus lacustris*, conical phytolith in epidermal cell (silica 3.5%). (E) *Carex secta*, silica in outer epidermal wall (silica 2.6%). (F) *Carex testacea*, silica in outer epidermal wall (silica 7.2%). Scale bars: A = 100 µm; B = 25 µm; F = 10 µm.

The results are summarized in Table 2, encompassing a comparatively wide range of silica‐bearing monocots, together with relevant comparative literature on silica deposition and cell‐wall‐bound ferulic acid in each family or order. Character optimizations are shown in Figure 2. There is a close association between silica deposition and the presence of cell‐wall bound ferulic acid (Figure 2D). The results highlight the distribution of silica phytoliths in monocots, which are restricted to orchids (Figures 1A–C, 3), and commelinids (Figures 4, 5, 6, 7, 8). Among non‐commelinid monocots, we found no observable silica deposition in leaves of Acoraceae (*Acorus gramineus*), the sister taxon to all other monocots, and only unlocalized traces and/or amorphous fragments of silica in the non‐commelinid orchid relatives *Astelia fragrans* (Asteliaceae) and *Borya sphaerocephala* (Boryaceae). Among these taxa, low levels of background silica were occasionally recorded (up to 1.5% in a leaf transverse section of *Acorus gramineus*, 1.2% in *B. sphaerocephala*), but there was no evident silica deposition as phytoliths.

We also found no silica deposition in leaves of scattered commelinids, many of them aquatics. Within Commelinales, silica deposits are reliably absent from the aquatic families Philydraceae and Pontederiaceae, and also from some Haemodoraceae, though granular silica is deposited in bundle sheath cells in some species of Haemodoraceae subfamily Conostylidoideae (Table 2). Silica deposition is also more or less absent from the aquatic or semiaquatic families of Poales (Eriocaulacaeae, Juncaceae, Mayacaceae, Typhaceae, and Xyridaceae). We found only occasional asymmetric fragments of silica in *Juncus* and only small unlocalized traces of silica in *Joinvillea plicata* (Figure 4E), though irregular silica deposits have been reported in both Joinvilleaceae and Juncaceae (Cutler, 1969; Tomlinson, 1969; Strömberg et al., 2016). We also found no trace of silica deposition in *Typha* or *Xyris*, as previously reported (Table 2).

### Diversity of phytoliths in monocots

Silica bodies that accumulate in the cell lumen are located either in the bundle sheath or in the epidermis, often associated with sclerenchyma. In contrast, cell‐wall bound silica is mostly epidermal (or hypodermal) and sometimes localized in stomata, trichomes, or papillae. Orchids possess lumen‐bound phytoliths that form inside bundle sheath cells, often occurring in axial rows, and always associated with sclerenchyma, either in vascular bundles or fiber bundles. Orchid phytoliths are always absent from the epidermis. Studies on orchids have shown that silica bodies—either conical or spherical—are present in the subfamilies Apostasioideae (Figure 1A) and Epidendroideae, but largely absent from Cypripedioideae, Orchidoideae, and Vanilloideae (Table 2). We found only unlocalized silica fragments in leaves of *Vanilla* (subfamily Vanilloideae), though background silica was recorded. *Dendrobium* (subfamily Epidendroideae) possesses silica bodies that are spherical with surface projections, but sometimes appear conical or flat‐sided before eruption from the enclosing membrane (Figure 3).

Similarly, all members of the palm family (Arecaceae) examined to date possess discrete solid lumen‐based silica bodies located only in bundle sheath cells (Figures 1, 4).

The family Dasypogonaceae also has silica bodies that accumulate in the cell lumen, either as solid bodies or as granular silica (Figures 1, 4), but they differ from Arecaceae in that silica is mostly epidermal or sometimes also present in the mesophyll, rarely in the bundle sheath or associated with sclerenchyma. In Zingiberales (Figure 5), silica is present in bundle sheath cells and rarely present in the epidermis. However, phytoliths are often relatively amorphous in this clade (see also Chen and Smith, 2013). Our EDX analysis of *Strelitzia* and *Hanguana* (Zingiberales) identified fragmented silica bodies in the cell lumen, entirely localized in the vascular bundle sheath cells in *Strelitzia* sp. (especially in hypodermal cells) but more widely dispersed across leaf tissues in *Hanguana* sp. In Commelinaceae (Commelinales), we found very high levels of silica in leaves, sometimes forming amorphous traces in the mesophyll, mainly located as dense granular deposits in the hypodermal layer (e.g., in *Pollia macrophylla*; Figure 5) and also in the epidermis, extending from the inner wall and forming a circular disc‐like canopy over the outer wall (in *Pollia condensata*, Figure 5).

The largest commelinid order, Poales, is the most diverse with respect to silica presence and location in cells and tissues (Figures 6, 7, 8). Some Poales possess dense spherical silica bodies restricted to bundle sheath cells (e.g., Flagellariaceae and Restionaceae, Figure 6). In two of the early‐divergent families of Poales, Bromeliaceae, and Rapateaceae (Figure 7), silica is present as tiny spherical bodies inside the lumen of epidermal cells, occasionally also in hypodermal cells. In Bromeliaceae, the walls of the epidermal cells have become thickened so that the cell lumen is occluded and the silica bodies are embedded in the thickened secondary wall, sometimes protruding outward on the surface (Figure 7E, F). In *Rapatea xiphoides* (Rapateaceae), we found small, solid silica bodies in epidermal cells overlying blocks of sclerenchyma, and also occasional larger (sometimes star‐shaped) bodies in the lumen of hypodermal cells underlying stomata (Figure 7A–D).

Amongst other Poales, grasses (Figure 6D) and sedges (Figure 8) possess phytoliths that are mostly epidermal, deposited either in the cell lumen or in the cell wall, often both. Our EDX analysis of Poaceae leaves found silica present as small bodies in epidermal cells overlying vascular bundles in *Glyceria maxima* (Figure 6D) and silica embedded in outer epidermal walls, especially in trichomes (in *Stipa gigantea*). In Cyperaceae, silica bodies that form in the epidermal lumen are often conical with a broad base that merges into an underlying sclerenchyma cell, as in *Carex depauperata* (Figure 8A, B). Grasses and sedges also possess silica that is integrated into the epidermal cell walls. In Cyperaceae, we found cell‐wall‐bound silica in most species, especially on the adaxial leaf surface in *C. secta* (Figure 8E) and C. *testacea*, Figure 8F). Cell‐wall‐bound silica is often densely localized in trichomes and papillae, especially in the papillae overarching the abaxial stomata in *Carex* and *Schoenoplexus lacustris* (Figure 8).

## DISCUSSION

### Distribution and diversity of silica deposition in monocots

Our investigation highlights the distribution of silica phytoliths in at least two of the five orchid subfamilies: the early‐divergent Apostasioideae and derived Epidendroideae, and in all commelinid orders (Arecales, Commelinales, Poales, and Zingiberales), plus the “wildcard” commelinid family Dasypogonaceae. In the context of modern phylogenies, and assuming secondary losses of this feature within both commelinids and orchids, this distribution indicates at least two independent evolutionary origins of silica phytoliths among monocot taxa, with secondary losses in some lineages.

It is noteworthy that high silica concentration, as documented in several investigations (e.g., Hodson et al., 2005; Song et al., 2020; Tombeur et al., 2023; Pang et al., 2025), is not necessarily correlated with the deposition of silica as phytoliths, either in the cell lumen or the cell wall. Several commelinids reliably lack phytoliths, including some Commelinales and Poales (listed in Table 2). Many of these taxa are aquatic or semiaquatic (e.g., Eriocaulaceae, Juncaceae, Mayacaceae, Philydraceae, Pontederiaceae, Typhaceae, and Xyridaceae). Yet, there is strong evidence for silica uptake in at least some of these taxa, and some reports have suggested silica present in tracheary elements (e.g., Piperno, 2006). For example, a relatively high (though variable) silica content is present among species of *Juncus* (Lanning and Eleuterius, 1978, 1981), which mostly lack phytoliths, as confirmed in our study. Furthermore, all of the aquatic commelinids that lack phytoliths possess cell‐wall bound ferulic acid, which is strongly correlated with silica deposition.

Why do so many aquatic commelinids lack phytoliths? One explanation for this apparent incongruity could be that silicic acid persists in supersaturated colloidal solution in the xylem sap in these taxa, without being deposited on the primary cell wall or in the cell lumen. Silicic acid occurs in diluted aqueous solutions, and the final step in silica body formation requires appropriately high concentrations and/or the presence of lignin. This explanation would correspond with the leaves of some of the submerged or emergent aquatic taxa, such as species of Eriocaulaceae, Mayacaceae, Pontederiaceae, and Typhaceae, which are aerenchymatous with large water‐filled intercellular spaces surrounding lobed mesophyll cells and relatively little sclerenchyma (Tomlinson, 1969; Splett et al., 1993; Sousa et al., 2016). In contrast, many emergent taxa, including Juncaceae, have strongly lignified aerial tissues. We found only occasional asymmetric fragments of silica restricted to the outermost mesophyll layers in the highly lignified leaves of *Juncus xiphioides*. The apparent absence of phytoliths in our material of *Joinvillea* (a non‐aquatic herb) merits further investigation; Strömberg et al. (2016) found silica deposition in two species of *Joinvillea*.

In the majority of commelinids, silica phytoliths are highly characteristic in appearance and located either in the bundle sheath or epidermis. Occasional fragmentary, amorphous or relatively disorganized deposits detected in the mesophyll could be at least partly attributable to the artefactual result of rapid drying of the leaves, especially among relatively high silica accumulators such as some Commelinales and Zingiberales (Hodson et al., 2005; Katz, 2015; Trembath‐Reichert et al., 2015). Fragmentary silica particles occur in the mesophyll in some Zingiberales, including *Hanguana* (Figure 5), which is semiaquatic, and also in *Strelitzia* and *Etlingera*. Similarly, in some Commelinaceae, in which most deposited silica is epidermal, we found occasional amorphous silica deposits in the mesophyll that could have precipitated during the drying process. Tomlinson (1966) identified different types of silica in epidermal cells in Commelinaceae leaves, either idioblastic (with silica bodies embedded in the inner epidermal wall) or relatively standard, with silica present in the unthickened outer wall. In *Pollia condensata* (Figure 5), silica is localized in the disc‐like central papillae of epidermal cells. We found no evidence of silica in fibers or tracheary elements, though some authors have reported silicification of these cell types, especially in fruits (e.g., in date palm and rice: George et al., 2020; Nakamura et al., 2021).

The shared presence in orchids and palms of essentially similar lumen‐bound phytoliths that form inside bundle sheath cells could indicate that this type represents the plesiomorphic condition among phytolith‐bearing monocots (Figure 2B), though rather similar spherical bodies occur in bundle sheath cells in some Poales, such as *Flagellaria* and *Restio* (Figure 6). However, this tentative inference of character evolution is highly sensitive to tree topology, since other analyses place Asparagales and Liliales as a sister pair, and relationships among the commelinid orders differ in recent analyses (Givnish et al., 2018; Timilsena et al., 2022; Zuntini et al., 2024).

The four genera of the “wildcard” family Dasypogonaceae were formerly assigned to the non‐commelinid family Xanthorrhoeaceae (Asparagales); they were transferred to the commelinid clade based partly on early molecular indications (Chase et al., 1995) that were strongly reinforced by morphological characters, including identification of silica bodies using EDX (Rudall and Chase, 1996) and detection of cell‐wall‐bound ferulates (Rudall and Caddick, 1994). However, within commelinids, the precise relationships of Dasypogonaceae remain uncertain (see also Rudall and Conran, 2012); some analyses strongly support a close association with palms (Timilsena et al., 2022), but in others they are variously placed as sister to Poales, sister to all other commelinids except Arecaceae, or sister to all other commelinids except Poales (Givnish et al., 2018; Zuntini et al., 2024). Dasypogonaceae differ from palms with respect to silica deposition (Figures 1, 4); silica is epidermal in Dasypogonaceae rather than present in the bundle sheath or associated with sclerenchyma, as in palms. Indeed, Dasypogonaceae remain a special case in this respect; they resemble Poales in possessing epidermal silica and resemble Zingiberales in that the phytoliths are often granular and rarely associated with sclerenchyma.

Of the 15 families of Poales, six deposit little or no silica (mostly aquatics: see discussion above); two deposit silica mostly or exclusively in the bundle sheath, two have mostly epidermal silica (often associated with underlying sclerenchyma), and the remainder deposit silica in both epidermal and subepidermal tissues (Figures 6, 7, 8). For example, the characteristic conical silica bodies of sedges are located within epidermal cells overlying sclerenchyma bundles; they develop by nucleation of silica onto lignified projections that extend from the thickened inner tangential walls of the epidermal cells (Mehra and Sharma, 1965). Rapid growth of the silicified process occurs simultaneously with wall thickening and lignification of the subepidermal sclerenchyma. A close association with sclerenchyma is particularly evident in Rapateaceae, in which the majority of phytoliths overlie axially oriented fiber bundles that form surface ridges (Figure 7A–D). Similarly, in Bromeliaceae, most phytoliths are located inside epidermal cells in which the lignified walls have compacted the cell contents (Figure 7E, F).

### Cell‐wall chemistry underpins silica deposition

Why are phytoliths confined to commelinids and orchids among monocots? At least for commelinids, the answer appears to lie in the particular phenolic acids present in the cell walls. There is an apparent association between silica deposition and cell‐wall‐bound ferulic acid (Figure 2D). The commelinid clade is unusually well characterized morphologically, not only by the shared presence of silica, but also by particular types of epicuticular wax and patterns of stomatal development and by the presence of ferulic acid in unlignified cell walls (Harris and Hartley, 1980; Rudall and Conran, 2012). Plant cell walls consist of cellulose arranged in tightly packed crystalline microfibrils, which binds with various macromolecules to form different complexes (Mnich et al., 2020). The primary cell walls of grasses and other commelinids contain phenolic compounds such as ferulic acid and *p*‐coumaric acid in a hemicellulose matrix containing mixed‐linkage glucans and glucuronoarabinoxylans (GAXs). As the leaf ages, the secondary cell walls accumulate lignin, a polymer that provides structural rigidity and hydrophobicity (Figueiredo et al., 2019; Zexer and Elbaum, 2020).

A close association between silica and lignin has long been reported: Silica bodies are most commonly located in cells positioned immediately adjacent to lignified tissues, either in the parenchymatous sheath of vascular bundles or in epidermal cells, often associated with underlying sclerenchyma, as our study demonstrates. Yet lignin is common in land plants, even those that lack silica deposition. Other studies in plants (especially grasses) have suggested a link with cell‐wall‐bound ferulic acid (Kulik et al., 2009; Nakamura et al., 2021; Zexer et al., 2023). Ferulic acid forms cross‐links with lignin in the cell wall, and both components appear to have a role in silica deposition (Zexer and Elbaum, 2020). During phytolith formation in *Sorghum* roots, complexes of arabinoxylan and ferulic acid provide nucleation sites for silicification, with a possible associated role for mixed‐linkage glucans (Soukup et al., 2017). In thick‐walled cells, such as the outer walls of epidermal cells, layers of cellulose can form a scaffold for organized polymerization of intrafibrillar silica nanoparticles, probably catalyzed by polysaccharides such as ferulated hemicellulose (Nakamura et al., 2021; Zexer et al., 2023).

Comparative studies using UV‐fluorescence microscopy have shown that the primary cell walls of all commelinid monocots examined to date contain ferulic acid (Harris and Hartley, 1976, 1980; Rudall and Caddick, 1994; Parr et al., 1996; Harris et al., 1997; Smith and Harris, 1999; Parker et al., 2003; Harris and Trethewey, 2010). The apparent absence of ferulic acid from the primary cell walls of all non‐commelinid monocots indicates that its presence is a synapomorphy for commelinid monocots (Figure 2). This character therefore broadly correlates with the occurrence of phytoliths, except in some aquatic commelinids (which lack phytoliths but possess ferulic acid), and in orchids (which contain phytoliths, at least in some species, but reportedly lack ferulic acid). Cell‐wall‐bound ferulic acid is otherwise relatively uncommon in land plants, except in many pteridophytes (e.g., *Equisetum* and many ferns), which contain relatively high levels of ferulic acid (Glass and Bohm, 1969; Fry et al., 2008); significantly, these taxa also produce phytoliths (Sundue, 2009; Mazumdar, 2011; Leroux et al., 2013; Trembath‐Reichert et al., 2015). Indeed, there are strong parallels between commelinid monocots and pteridophytes in terms of silica deposition. In the fern *Adiantum raddianum*, which has thick‐walled silica cells in the epidermis, silica is deposited in the outer zone of the primary cell wall and the cutinized region outside it, but not in the secondary cell wall or the cell lumen (Leroux et al., 2013). This clear localization in the cell wall indicates that silica deposition occurs during early development.

In contrast, orchids reportedly lack ferulic acid in their primary cell walls, though this non‐commelinid family would merit further investigation for both silica and hemicellulose composition because existing data are sometimes contradictory. In orchids, silica phytoliths are present in the largely tropical subfamilies Apostasioideae and many genera of Epidendroideae, but absent from the other three subfamilies, Cypripedoidoidae, Orchidoideae, and Vanilloideae (Møller and Rasmussen, 1984), which include both tropical and temperate taxa. In contrast, Pang et al. (2025) reported that Apostasioideae and Cypripedoidoidae are silica‐rich and the other subfamilies silica‐poor. Our EDX analysis of *Dendrobium* (Epidendroideae) found small phytoliths in relatively high numbers but restricted to bundle sheath cells, so that overall silica levels are relatively low across the leaf (Figure 3). Hodson et al. (2005) reported low silica accumulation in *Bletilla* (a temperate genus of Epidendroideae), though *Bletilla* possesses silica bodies in bundle sheath cells (Møller and Rasmussen, 1984). Unfortunately, relatively little is currently known about phenolic acids in orchid leaves, though Harris and Hartley (1980) reported a negative result for ferulic acid in the four orchid species that they sampled, specifically, one species of Orchidoideae (*Anacamptis pyramidalis*), in which phytoliths are absent, and three species of two genera of Epidendroideae (*Coelogyne* and *Cymbidium*), in which phytoliths are present. Yet studies on *Vanilla* (which lacks phytoliths) indicate that vanillin, a flavor compound that is harvested from *Vanilla* pods, is formed by direct conversion from ferulic acid (e.g., Gallage et al., 2014), suggesting that ferulic acid could be present in some tissues at early developmental stages.

### Phytolith shapes are determined during silica deposition

For the silica‐bearing taxa, our results highlight taxonomic similarities and differences in modes of deposition, including phytolith location and morphology. Colloidal chemistry is key to this process; biogenic silica deposition passes through a colloidal phase, and cellulose microfibrils attract colloids of silica (Hemsley et al., 1994; Zexer et al., 2020, 2023). Using silica colloids as precursors, Ho et al. (2007) developed a method to synthesize mesoporous silica spheres that were remarkably similar to biogenic silica bodies that we have observed in monocots (e.g., in *Restio*, Figure 6F). Condensation at higher concentrations forms a colloidal solution, and further polycondensation can lead to particle aggregation (Lopez et al., 2005). Phytoliths that fill the cell lumen are generally formed late in cell maturation, whereas cell‐wall‐bound silica is deposited initially in the primary cell wall. During the formation of dumbbell‐shaped silica cells in the leaf epidermis of rice, cell‐wall lignification and silicification precede silica hyperaccumulation within a progressively supersaturated cell lumen, resulting in a silica body that is structurally consistent with amorphous silica (Zhang et al., 2013).

The presence of only lumen‐based silica bodies in many taxa could also be related to differences in cell‐wall chemistry (see also Hodson, 2016, 2019). Smith and Harris (1999) found relatively small proportions of pectic rhamnogalacturonans in the unlignified cell walls of some Poales, including Restionaceae and Flagellariaceae, which possess discrete silica bodies in bundle sheath cells, but also in Anarthriaceae and Ecdeiocoleaceae, in which silica deposition is low or absent (Table 2).

Orchid silica cells were termed stegmata or cover cells (German *deckzellen*) by early researchers because they occur only in the sheathing cell layer that encloses (or covers) a vascular bundle or a sclerenchyma bundle (Kohl, 1889). These cells possess a thin outer wall and thickened inner wall adjacent to the associated fibers. The wall thickening extends around the sides of the cell, is initially cellulosic, and later becomes lignified, but mostly remains unsilicified. Orchid stegmata are discernible in young leaves even before silica deposition, indicating that they are pre‐programmed for phytolith formation (Zhou, 1995). Similarly, in grasses, regular lignification of the walls of specialized silica cells precedes silica deposition in the lumen to form their distinctive silica bodies (Zhang et al., 2013).

Both the mode and site of deposition together determine the shape of the phytolith. Silica deposition is either associated with cell walls, scaffolded by organic materials, or an organic backbone is lacking or (more likely) tenuous or short‐lived, so that the solid silica body grows by hyperaccumulation in a progressively supersaturated vacuole (Guerriero et al., 2016; Soukup et al., 2017; Bokor et al., 2019). Both mechanisms are potentially templated by the presence of lignin, and both are often associated with sclerenchyma (Zhang et al., 2013; Soukup et al., 2017).

Epidermal silica can sometimes occur as minute bodies embedded inside chambers that represent the remains of cells in which the lumen has become increasingly occluded by wall thickening. This condition occurs in Bromeliaceae and Rapateaceae, which are placed among the early‐divergent families of Poales in molecular phylogenetic analyses. In these two families, regular rows of minute silica bodies are located above sclerenchyma strands (Figure 7), becoming enclosed into chambers within the thickened epidermal cell wall. Despite reports of relatively low silica accumulation in Bromeliaceae (Hodson et al., 2005), such minute epidermal silica bodies are very common in this family (Tomlinson, 1969).

### Cell‐wall‐bound silica occurs as an integrated layer in many grasses and sedges

In addition to silicified papillae and trichomes, a more continuous near‐surface silica layer is present across the outer epidermal wall in many Cyperaceae and Poaceae (Figures 6, 8), both of which also possess epidermal silica bodies in the cell lumen over hypodermal sclerenchyma cells. In these two families, silica deposition can also occur in the walls of specialized epidermal cells such as stomata and prickle hairs, but also as a continuous layer in some taxa (see also Mehra and Sharma, 1965; Metcalfe, 1971; Soni et al., 1972; Estelita and Rodrigues, 2012). In grasses, this type of organization has been termed a cuticle‐silica double layer or a silica‐cellulose membrane (Yoshida et al., 1959, 1962a, 1962b), forming a matrix‐bound silica layer that might enhance desiccation tolerance, provide resistance to predation by insects and pathogens, and help to maintain cell‐wall structure (Yoshida et al., 1962a, b; Sakai and Thom, 1979; He et al., 2015). The relatively high levels of silica integrated into grass and sedge cell walls could be related to relatively high concentrations of ferulic acid and mixed‐linkage glucans in these taxa (Harris and Trethewey, 2010). In Commelinaceae, which includes taxa that accumulate some of the highest levels of silica, a significant proportion of the silica is incorporated into the trichomes and outer epidermal wall, sometimes as distinct hat‐shaped structures with a central papilla (Figure 6D). As Tomlinson (1966) commented, more detailed comparative studies are needed to understand phytolith structure and development in Commelinaceae.

### Diverse deposition patterns indicate differing functions and complex evolution

Biosilicification is widely (but sparsely) distributed among land plants. Apart from commelinid monocots and orchids, phytolith‐bearing taxa include some liverworts (e.g., *Mizutania*: Pressell et al., 2011), lycophytes (e.g., *Selaginella*: Lopes and Feio, 2020; Shih et al., 2022), *Equisetum* (Sapei et al., 2007; Law and Exley, 2011), many ferns (Sundue, 2009; Mazumdar, 2011), and a few magnoliids and eudicots (Postek, 1981; Giordano et al., 2019; Zexer et al., 2023). Our study underscores the broader potential for phytolith analysis in the context of palaeoecology (Brightly et al., 2024; Siver et al., 2025). Other investigations have focused on functional aspects of silica biology across these taxa (e.g., Strömberg et al., 2016). Cell‐wall‐bound silica can help to maintain the rigidity of the aerial organs and also provide resistance to pathogens and herbivores (Sakai and Thom, 1979; Zhang et al., 2013; Soukup et al., 2017). Indeed, silicified trichomes and papillae are common among commelinid monocots (De Bary, 1884; Tomlinson, 1966; Zancajo et al., 2019; Hall et al., 2020). Pang et al. (2025) suggested that silica deposition in epidermal cell walls could improve temperature regulation and water balance and potentially have a cooling effect, helping to regulate stress; high silica uptake and deposition could have evolved during warming paleoclimatic periods. However, this hypothesis remains difficult to test in monocots, given the ambiguities of detailed relationships among monocot clades.

## CONCLUSIONS

Although silica inclusions in plant tissues have long been documented, earlier comparative investigations were restricted by available technology and phylogenetic context. Most previous comparative studies either used chemical methods to detect silica or microscopy to identify silica bodies in the cell lumen based on their non‐crystalline appearance and characteristic shapes. However, plant‐based silica lacks intrinsic birefringence and is therefore most effectively visualized using energy‐dispersive x‐ray spectroscopy (EDX/EDS), especially deposits that are integrated into the cell wall. Our study is the first to use this technique using a comparative mapping approach with a phylogenetically broad sample and the first to explore silica distribution among tissues across the leaf or stem.

Our exploration of the diverse strands of literature on plant silica biology leads us to hypothesize that the restricted phylogenetic distribution of monocot phytoliths is at least partly attributable to cell chemistry, especially the inclusion of ferulic acid in primary cell walls, though this association apparently does not apply to orchids. We also note that documented high silica concentrations are not necessarily correlated with the deposition of silica as phytoliths, either in the cell lumen or the cell wall, perhaps indicating that silicic acid persists in high concentrations in the xylem sap in some taxa, especially among aquatic commelinids. We speculate that programmed variations in cell‐wall chemistry at different developmental phases could account for consistent differences in silica deposition among the silica‐bearing monocots. Our broad combinatorial approach using comparative EDX/EDS data on controlled silicification in monocots not only highlights the appealing aesthetic of these diverse biogenic constructions, but also allows us to place them in a modern phylogenetic and developmental context.

## AUTHOR CONTRIBUTIONS

P.J.R. designed and implemented the project, collected evidence, and led the writing of the manuscript. J.L. managed the lab procedures and helped with resources. M.K.M. helped with preparing material and directed methodology and operation of the SEM and EDX/EDS. All authors contributed to the writing and editing of the manuscript.

## CONFLICT OF INTEREST STATEMENT

The authors declare no conflict of interest.

## Data Availability

All relevant data are presented in the paper.
